# EDL6D, a bioactive peptide phenocopying SHBG-associated metabolic effects: a new preclinical lead compound for treating the metabolic dysfunction-associated fatty liver disease

**DOI:** 10.1186/s12967-026-08305-9

**Published:** 2026-05-16

**Authors:** Anna Alvarez-Guaita, Julia Cabrera-Serra, Beatriz Perez-Gonzalez, Maria Teresa Salcedo-Allende, Lidia Fuertes-Rioja, Lorena Ramos-Perez, Cristina Hernandez, Rafael Simó, David M. Selva

**Affiliations:** 1 Diabetes and Metabolism Research Unit, Vall d’ Hebron Institut de Recerca (VHIR), Universitat Autònoma de Barcelona and CIBERDEM (ISCIII), Pg Vall d’Hebron 119-129, Barcelona, 08035 Spain; 2 Endolipid Therapeutics S.L, Vall Hebron Institut de Recerca (VHIR), Barcelona, Spain; 3 Human Pathology Department, Vall d’Hebron University Hospital, Barcelona, Spain

**Keywords:** SHBG, Fatty liver, Fibrosis, Peptide, Lipogenesis, Triglycerides, Inflammation

## Abstract

**Background:**

Low plasma sex hormone–binding globulin (SHBG) levels are commonly observed in metabolic dysfunction–associated fatty liver disease (MASLD), a spectrum of disorders ranging from simple hepatocellular steatosis to steatohepatitis, fibrosis, and irreversible cirrhosis. We have previously demonstrated that SHBG overexpression reduces hepatic lipid accumulation by inhibiting lipogenesis both in vitro and in vivo.

**Methods:**

In the present proof-of-concept study, we have developed a bioactive peptide that phenocopies SHBG-associated metabolic effects (EDL6D) and have evaluated its ability to inhibit lipogenesis in vitro using HepG2 cells treated daily with 30 mM fructose. In addition, we assessed the preventive and therapeutic effects of EDL6D in vivo in wild-type mice with MASLD induced by a high-fat diet combined with 30% fructose in drinking water.

**Results:**

Treatment with EDL6D exerted both preventive and therapeutic effects, significantly reducing hepatic fat accumulation and promoting regression of MASLD compared with vehicle-treated mice. Mechanistically, EDL6D downregulated the mRNA expression and protein levels of key lipogenic enzymes, including ATP-citrate lyase, acetyl-CoA carboxylase, and fatty acid synthase, as well as peroxisome proliferator–activated receptor gamma, a central lipogenic transcription factor. Notably, EDL6D prevented the development of mild fibrosis by reducing the expression of collagen type I alpha 1 chain (Col1a1) and transforming growth factor beta 1 (TGF-β1).

**Conclusions:**

MASLD remains as an unmet medical need and disease progression to fibrosis represents a major clinical challenge. This study identifies EDL6D as a novel preclinical lead compound that targets both hepatic lipid accumulation and suggests an antifibrotic effect. These findings support the translational potential of bioactive peptides phenocopying SHBG for the treatment and prevention of MASLD.

**Supplementary Information:**

The online version contains supplementary material available at 10.1186/s12967-026-08305-9.

## Introduction

Steatotic liver disease (SLD) is one of the major causes of fatty liver disease, occurring when fatty acids are deposited in the liver and it is not as result of alcohol intake [1]. SLD represents a spectrum of diseases ranging from hepatocellular steatosis that can evolve to steatohepatitis and fibrosis leading to irreversible cirrhosis [2, 3]. The metabolic dysfunction-associated to steatosis (MASLD) is the first stage of the disease that can progress to a metabolic dysfunction-associated to steatohepatitis (MASH) [4]. The prevalence of MASLD is affecting up to 30–40% of adults globally, strongly linked to metabolic risk factors such as type 2 diabetes and obesity [5, 6].

Several studies have shown that obese subjects, type 2 diabetic (T2D) patients, and individuals suffering MASLD have low sex hormone-binding globulin (SHBG) levels [7]. SHBG is a glycoprotein secreted by the human liver into the blood where it binds and transports androgens and estrogens with high affinity regulating their bioavailability [8]. During the last decade, we have determined the molecular mechanism by which SHBG plasma levels are reduced in MASLD or MASH. Our studies have demonstrated that hepatic SHBG production is reduced by high carbohydrate diets (associated with hepatic lipid accumulation) by reducing hepatocyte nuclear factor alpha (HNF4α) levels [9], the most important transcription factor activating SHBG gene expression [10]. In addition, we have shown that pro-inflammatory cytokines such as tumor necrosis factor alpha (TNFα) and interleukin 1 beta (IL1β) are also able to reduce hepatic SHBG production [11–13], whereas anti-inflammatory cytokines such as adiponectin exert an upregulating effect [14]. This is important to explain the reduction of circulating SHBG levels observed in obesity and T2D [15]. Moreover, we have recently described that the transforming growth factor beta 1 (TGF-β1), a key inflammatory cytokine involved in the progression of MASH to fibrosis [16–18] and increased in MASH patients with fibrosis [19–22], is able to reduce hepatic SHBG production [23].

We have also addressed and highlighted the importance of the SHBG downregulation in MASLD patients, since we have shown that overexpression of human SHBG resulted in the reduction of lipid accumulation in the liver in two different mouse models of MASLD: a genetic mouse model (SHBG-C57BL/ksJ-db/db) and a diet induced mouse model (high fructose diet) [24]. The SHBG overexpression significantly reduced liver fat accumulation by reducing key lipogenic enzymes such as ATP-Citrate Lyase (ACLY), Acetyl-CoA Carboxylase (ACC) and Fatty Acid Synthase (FAS) as well as peroxisome proliferator activated receptor gamma (PPARγ), a key lipogenic transcription factor [24]. Importantly, the cell autonomous effects of SHBG on hepatic lipogenesis were demonstrated in HepG2 cells by modulating SHBG expression and by treating with exogenous SHBG [24]. Overall, these results suggest that the downregulation of SHBG induced by obesity and T2D lead to an increased hepatic lipogenesis which contributes to the development of MASLD. Therefore, SHBG can be contemplated as a therapeutic target to prevent and/or treat MASLD rather than a merely biomarker. However, the eventual reduction of sex steroid availability as a result of an enhancement of SHBG is limiting factor for its use in clinical practice. The development of bioactive peptides phenocopying SHBG-associated metabolic effects maintaining SHBG beneficial actions in the setting of MASH but without capacity of binding to sex steroids would overcome this problem.

Recent advances in metabolic and therapeutic research highlight the relevance of targeting the hepatic metabolic regulation and the cellular homeostasis in MASLD/MASH. In this regard, lysine acetylation has emerged as a key post-translational modification linking metabolic sensing and inflammation, two processes that are dysregulated in liver diseases [25]. In addition, increasing interest has been directed towards bioactive natural compounds with cytotoxic and antimicrobial properties which have shown the capacity to modulate oxidative stress and inflammation in metabolic disorders [26]. It is also important the development of innovative drug delivery systems, such as bilosome-based formulations enhancing the bioavailability of therapeutic agents in liver-associated diseases to improve efficacy of the treatments and minimize the side effects [27].

In this context, we developed EDL6D considering the results obtained by Khan and collaborators, that identified a small region of the human SHBG sequence with the capacity to bind to membranes [28]. Briefly, we synthetized several small D-peptides between 10 and 30aa containing these small region (further information can be found in the EP2024/065136). After the in vitro testing on anti-lipogenic, anti-inflammatory, anti-fibrotic and lipolytic activities of these peptides EDL6D was selected.

In the present proof-of-concept study, we evaluated the ability of a single dose of EDL6D to inhibit lipogenesis in vitro using fructose-treated HepG2 cells and to exert preventive and therapeutic effects in vivo in a diet-induced mouse model of MASLD. The experimental conditions and outcome measures were selected based on prior evidence from the literature and previous work addressing hepatic lipid metabolism and fibrosis progression. Our findings suggest that EDL6D is a novel preclinical lead compound for the prevention and treatment of MASLD.

## Results

### EDL6D treatment blocked the increase in key lipogenic enzymes (ACC, ACLY, FAS) and PPARγ induced by fructose in HepG2 cells

**Fig. 1:**
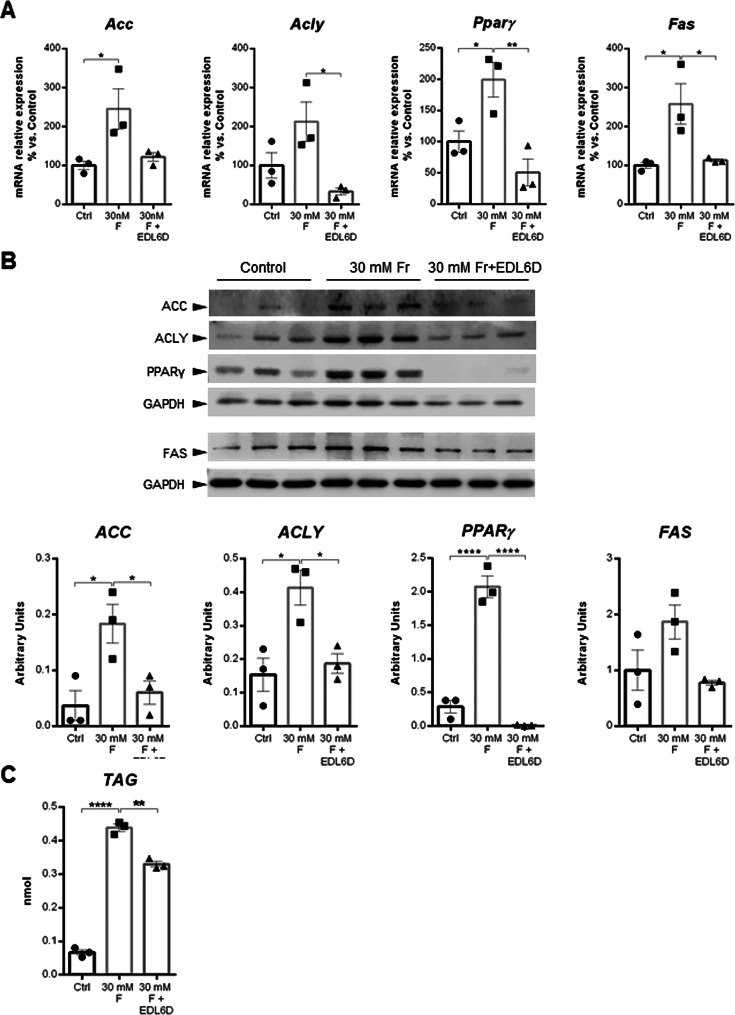
HepG2 stable cell line cultured cells control (Ctrl), treated with 30 mM fructose in the media or treated with 30 mM fructose plus (40 µg/ml) EDL6D (30 nM F+EDL6D) during 24 h. **A**. mRNA expression of Acetyl-CoA carboxylase (Acc), ATP-citrate synthase (Acly), Fatty acid synthase (Fas) and Peroxisome proliferator-activated receptor gamma (PPARγ) relative to control HepG2 cells. **B**. Representative western blot (WB) images and band quantification of ACC, ACLY, FAS and PPARγ of HepG2 cell lysates under the different treatments relative to Ctrl (*n* = 3). **C**. Triacylglyceride quantification in HepG2 lysates (*n* = 3). Data is presented as mean ± SEM of at least three experiments performed in triplicates and analysed by 1-way ANOVA with Bonferroni multiple comparison post hoc testing for all parametric data. *p < 0.05, **p < 0.01, ***p < 0.001, ****p < 0.0001

To study the effects of EDL6D on lipogenesis we first examined the effects of daily EDL6D (40 ng/ml) treatment on key lipogenic enzymes Acc, Acly, Fas and Pparγ in HepG2 cells treated in the presence or absence of fructose (30 mM) for five days. The results showed that treatment with fructose (30 mM) treatment induced a significantly increase in mRNA levels of Acc, Acly, Fas and Pparγ when compared with control HepG2 cells (Fig. 1A). Remarkably, in HepG2 cells treated with fructose (30 mM), the treatment with EDL6D blocked significantly the increase in mRNA levels of ACLY, ACC, FAS and PPARγ (Fig. 1A). These results were corroborated at protein level (Fig. 1B). Finally, EDL6D treatment was able to reduce the triglyceride (TG) accumulation induced by 30 mM fructose in HepG2 cells (Fig. 1C).

### Preventive effects of EDL6D treatment in the development of MASLD

**Fig. 2:**
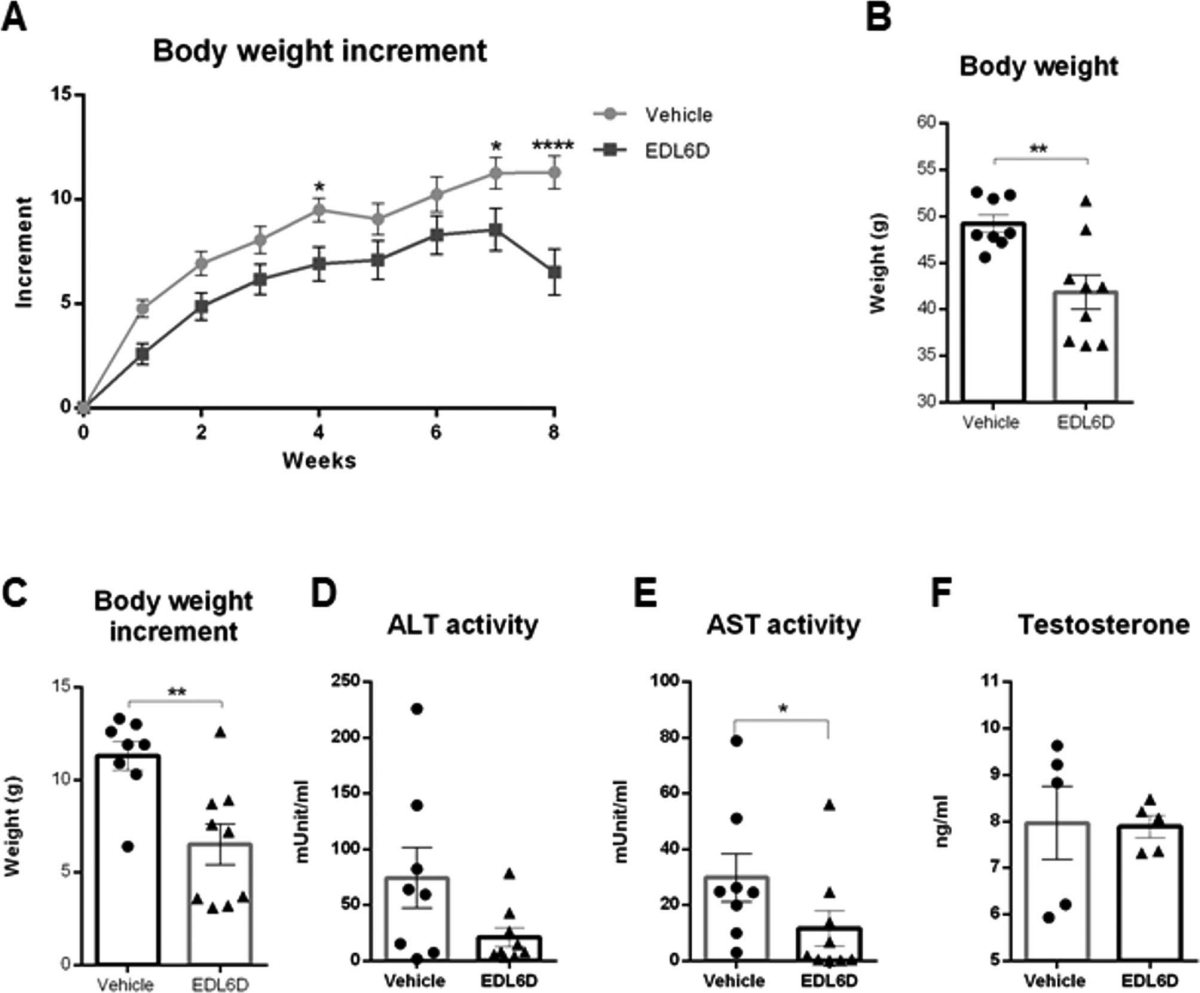
**A**. Weekly body weight gain of WT daily treated with vehicle/EDL6D mice over 8 weeks fed 45% HFD + 30% fructose in the drinking water (*n* = 7–9). **B** and **C**. Body weight and weight increment, respectively at the end time point (8 weeks) (*n* = 7–9). **D** and **E**. Alanine aminotransferase (ALT) and aspartate aminotransferase (AST) activity in vehicle and EDL6D treated mice serum samples at the end time-point (*n* = 7–9). **F**. Testosterone serum levels at the end of the experiment (*n* = 5). Data is presented as mean ± SEM and analysed by Two-way ANOVA and Bonferroni multiple comparison post hoc testing (**A**), t-Student test for parametric data (**B**, **C** and **F**) or Mann-Whitney for non-parametric data (**D** and **E**). *p < 0.05, **p < 0.01, ***p < 0.001, ****p < 0.0001

We next analyzed the in vivo effect of the EDL6D peptide over an 8-week period of mice fed with 45% high-fat diet (HFD) and 30% fructose in the drinking water (HFD + 30%Fr), treated in parallel with either vehicle or EDL6D (Fig. 2A). The weekly body weight monitoring showed that daily treatment with EDL6D was able to significantly reduce the total body weight gain when compared with vehicle treated mice at the end of the 8-week period (Fig. 2A-B). Moreover, the EDL6D treatment was able to reduce significantly the total body weight when compared with vehicle treated mice (Fig. 2C). The EDL6D reduction in total weight gain could be attributed to a reduction in the total weight of liver, brown adipose tissue and subcutaneous, inguinal and epididymal adipose tissue (Table 1). Remarkably, EDL6D treatment did not alter food consumption when compared with vehicle treated mice (Supplementary Fig. 1A).

**Table 1:** Tissue weight preventive study

Tissue	Vehicle	EDL6D
Liver (g)	2.46 ± 0.15	1.87 ± 0.14*
Hepatic Index (%)	4.68 ± 0.42	4.43 ± 0.17
Kideny (g)	0.47 ± 0.02	0.46 ± 0.01
Spleen (g)	0.11 ± 0.01	0.10 ± 0.004
Testis (g)	0.21 ± 0.01	0.22 ± 0.01
Brown Adipose	0.43 ± 0.04	0.23 ± 0.03***
Epididymal Adipose	2.44 ± 0.08	1.93 ± 0.09***
Inguinal Adipose	1.70 ± 0.09	1.16 ± 0.18*

Liver, kidney, spleen, testis, brown adipose tissue and epididymal adipose tissue weight in Ctrl, vehicle and EDL6D treated mice at the end time point. Hepatic index percentage is calculated respect body weight at the end of the experiment (*n* = 8–10). Data is presented as mean ± SEM and analysed by t-Student test. *p < 0.05, **p < 0.01, ***p < 0.001, ****p < 0.0001

EDL6D treatment tend to reduce the non-specific liver damage measured as alanine aminotransferase (ALT) and aspartate aminotransferase (AST) activity in serum, however the differences were not significant (Fig. 2D-E, respectively). Importantly, EDL6D treatment did not affect testosterone serum levels when compared with vehicle treated mice (Fig. 2F).

**Fig. 3:**
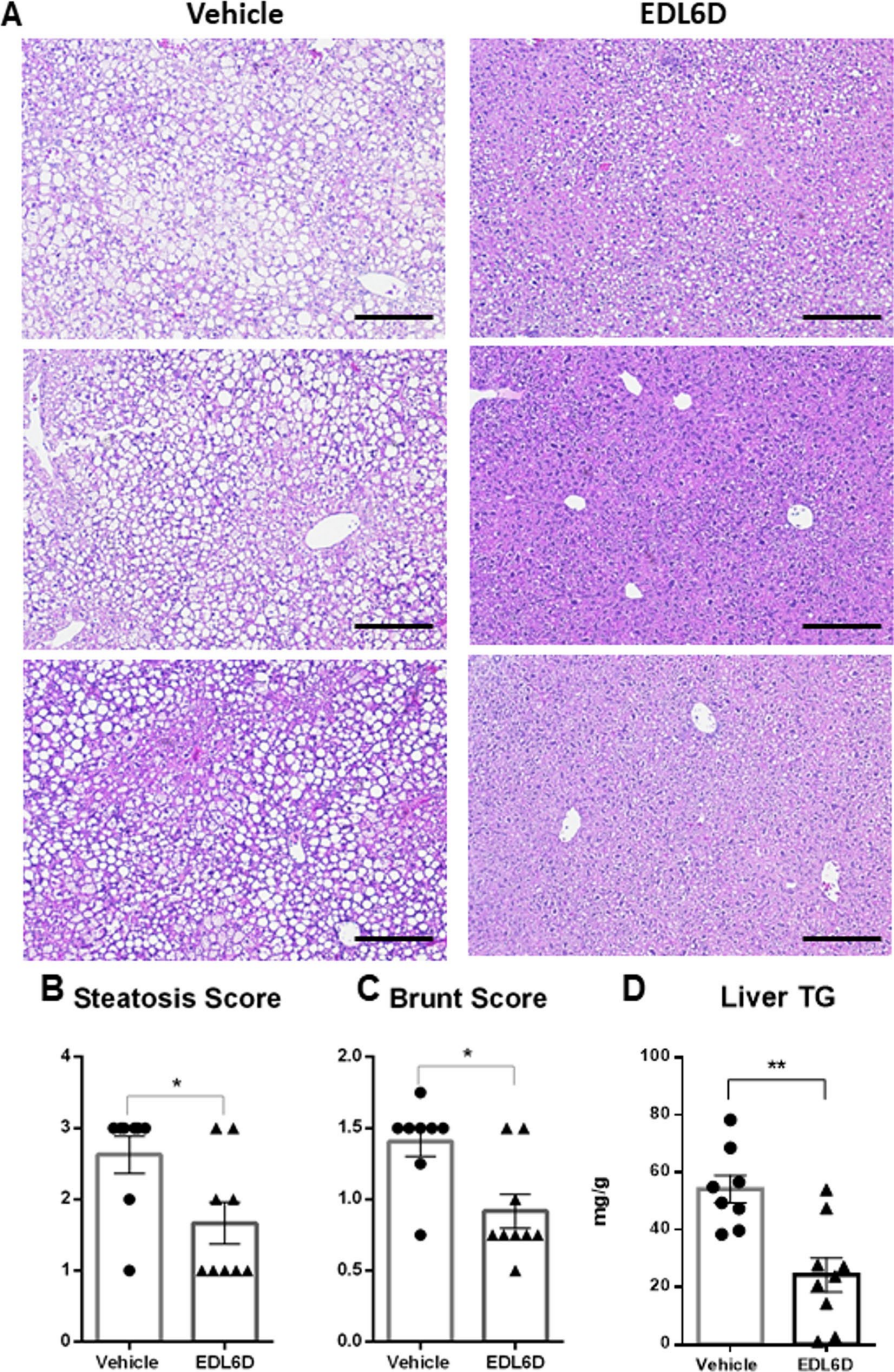
**A**. Representative images (10X – 100 μm scale bar) of hematoxylin/eosin staining of paraffin embedded liver tissue from vehicle and EDL6 treated mice upon 8 weeks 45% HFD + 30% fructose in the drinking water (*n* = 7–9). **B**. Liver steatosis qualitative quantification (Steatosis Score) (from 0 until 4) and **C**. Liver Brunt Score (from 0 until 4) from Vehicle and EDL6D treated livers at the end time point performed by an external hepatologist (*n* = 7–9). **D**. Triacylglyceride quantification of liver lysates at the same time point (*n* = 7–9). Data is presented as mean ± SEM and analysed by Mann-Whitney for non-parametric data. *p < 0.05, **p < 0.01, ***p < 0.001, ****p < 0.0001

The liver histological examination at the end time-point revealed that mice treated with vehicle developed a clear hepatic steatosis, whereas EDL6D treated mice did not (Fig. 3A). Importantly, the protective effect of EDL6D was confirmed by an external double-blinded analysis of the hematoxylin/eosin staining sections performed by an experienced pathologist and calculating the steatosis and brunt scores (Fig. 3B-C). The reduction in steatosis was further corroborated by measuring the triacylglyceride (TG) content of livers in vehicle and EDL6D treated mice. Accordingly, the results showed that mice livers treated with EDL6D had significantly lower TAG content (Fig. 3D), while there was no significant difference in tissue cholesterol levels (Supplementary Fig. 1C).

**Fig. 4:**
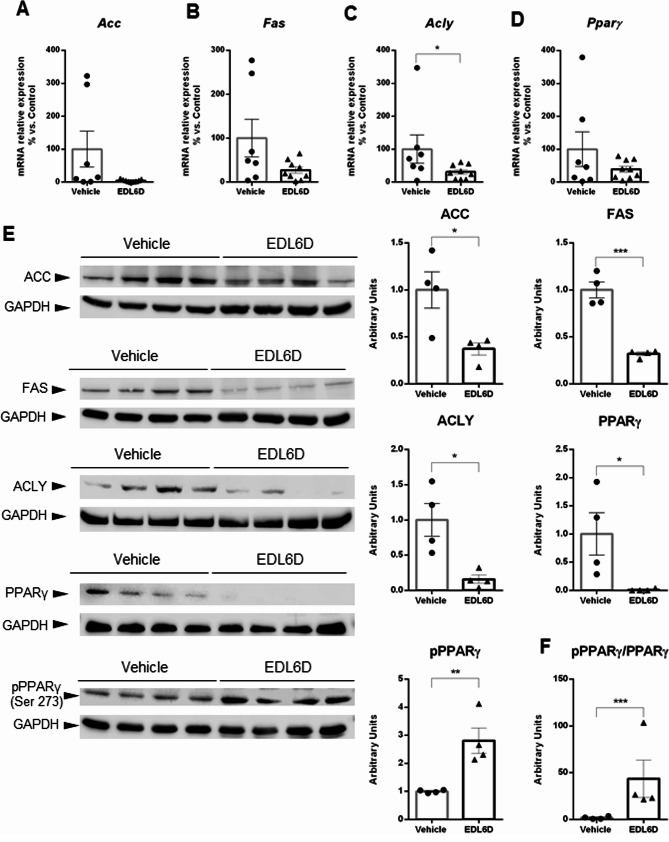
**A**, **B**, **C** and **D**. mRNA expression of *Acc*, *Acly*, *Fas* and *Pparγ* relative to vehicle treated mice livers (*n* = 7–9) and representative western blot (WB) images of ACC, ACLY, FAS, PPARγ and pPPARγ and its respective quantifications compared to vehicle group (*n* = 4). F. pPPARγ/PPARγ ratio quantification (*n* = 4). Data is presented as mean ± SEM and analysed by Mann-Whitney for non-parametric data (**A**, **B**, **C** and **D**) and t-Student test for parametric data (**E** and **F**). *p < 0.05, **p < 0.01, ***p < 0.001, ****p < 0.0001

**Fig. 5:**
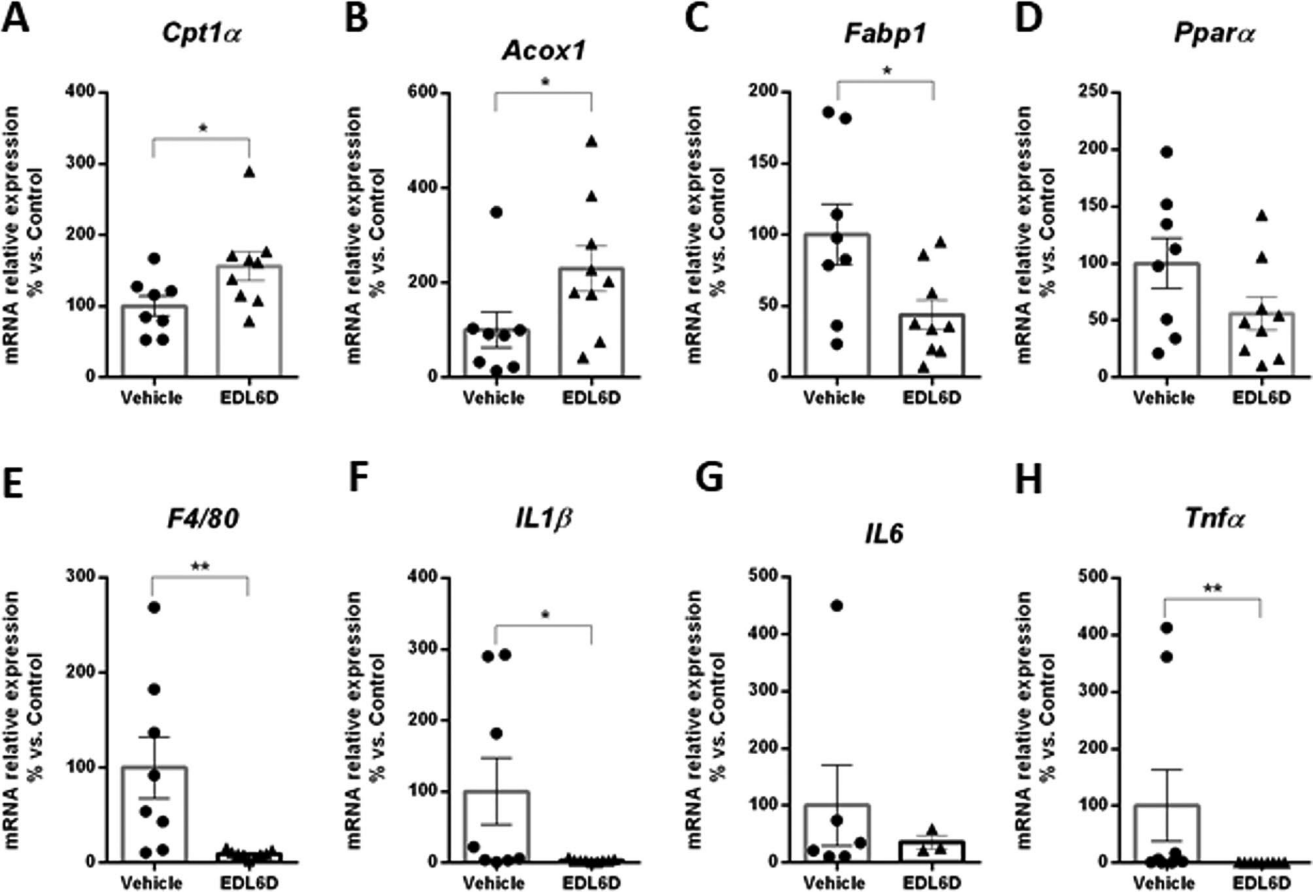
**A**-**K** mRNA expression of *Cpt1α Acox*,* Pparα*,* Fabp1*,* F4/80*, *Il1β*, *Il6* and *Tnfα*, relative to vehicle treated mice livers (*n* = 7–9). Data is presented as mean ± SEM and analysed by Mann-Whitney for non-parametric data (**B**, and **H**) and t-Student test for parametric data (**A**, **C**, **D**, **E** and **G**). *p < 0.05, **p < 0.01, ***p < 0.001, ****p < 0.0001

In order to elucidate the molecular mechanisms by which EDL6D treatment prevents hepatic steatosis we decided to analyze the hepatic levels of key genes involved in hepatic *de novo* lipogenesis (*Acc*, *Acly*, *Fas* and *Pparγ*), β-oxidation (*Cpt1α*, *Acox1*, *Ppar*α), fatty acid uptake (*Fabp1*). The analysis of the hepatic *de novo* lipogenesis key proteins ACC, ACLY, FAS and PPARγ mRNA and protein from these mice revealed that EDL6D treatment was able to reduce the mRNA and protein levels of *Acc* (Fig. 4A), *Acly* (Fig. 4B), *Fas* (Fig. 4C) and *Pparγ* (Fig. 4D) when compared with mice fed HFD + 30%Fr and treated with vehicle, however significance was only reached at protein level (Fig. 4E). Importantly, EDL6D treatment was able to increase the PPARγ phosphorylated protein levels when compared with vehicle treated mice (Fig. 4E), an this was also shown by the pPPARγ/PPARγ ratio (Fig. 4F). The expression of the β-oxidation carnitine palmitoyltransferase I α (*Cpt1α*) and acyl-coenzyme A oxidase 1 (*Acox1*) revealed that EDL6D treatment was able to increase the mRNA levels reaching significance for *Cpt1α* (Fig. 5A and B, respectively). Moreover, fatty acid uptake gene Fatty Acid Binding Protein 1 (*Fabp1*) was downregulated in the peptide treated mice compared to vehicle (Fig. 5C), while *Pparα* mRNA levels were decreased in the EDL6D treated mice (Fig. 5D).

Next, analysis of the related inflammation gene expression revealed that EDL6D treatment was able to prevent the increase of mRNA levels of *F4/80*, a marker of macrophage infiltration, and proinflammatory cytokines such as interleukin 1 beta (*Il1β*), interleukin 6 (*Il6*) and tumor necrosis factor alpha (*Tnfα*) induced by HFD + 30%Fr (Fig. 5E-H).

### Therapeutic effects of EDL6D treatment in stablished MASLD

**Fig. 6:**
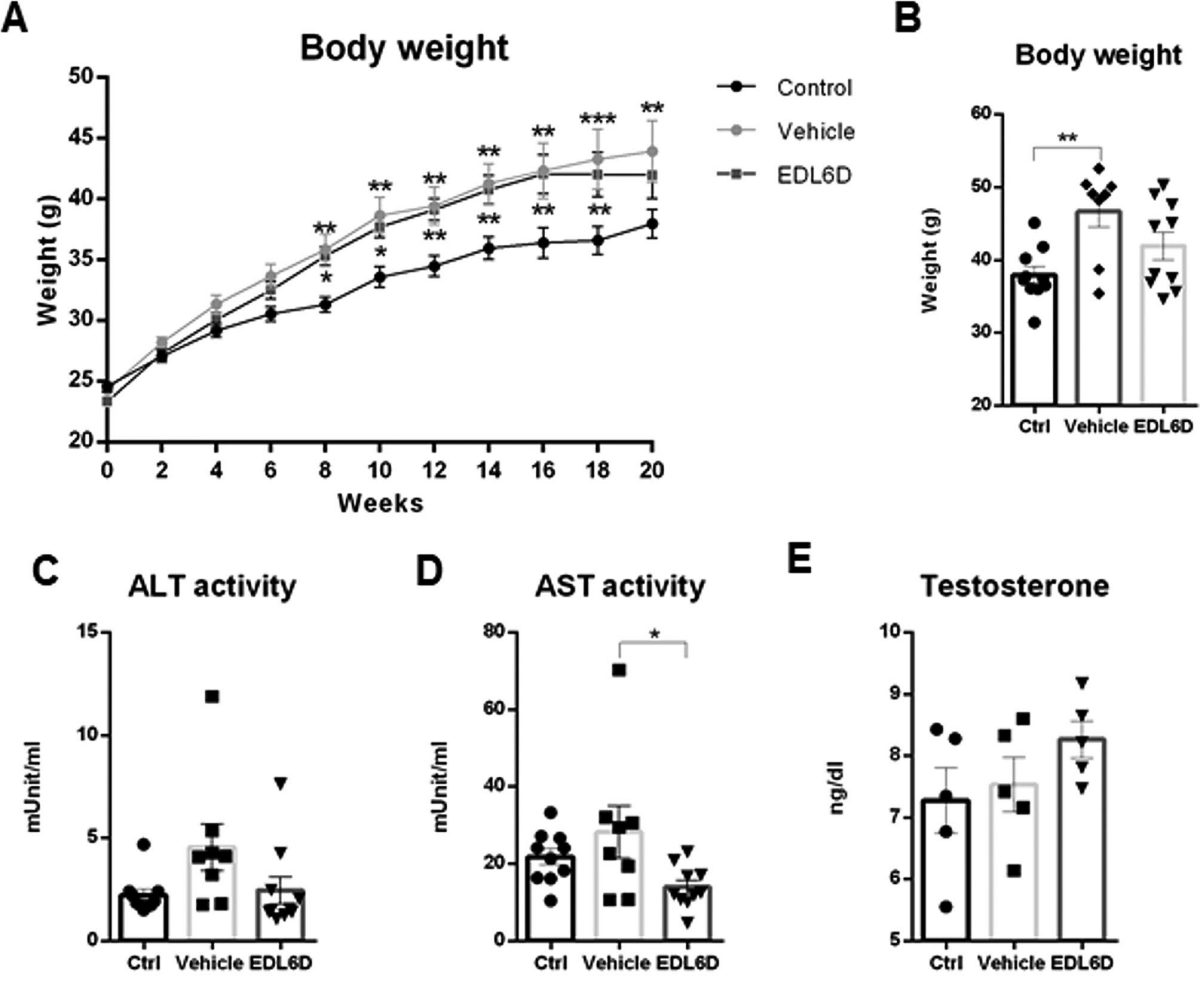
**A**. Every 2-days body weight gain of WT fed chow diet or 45% HFD + 30% fructose in the drinking water, daily treated with vehicle (Ctrl and Vehicle)/EDL6D over 10 weeks after 10 weeks fed either chow or high-fat diet, respectively (*n* = 8–10). **B** and **C**. Body weight and weight increment at the end of the experiment (*n* = 8–10). **D** and **E**. Alanine aminotransferase (ALT) and aspartate aminotransferase (AST) activity in Ctrl, vehicle and EDL6D treated mice serum samples at the end time-point (*n* = 8–10). **F**. Testosterone serum levels at the end of the experiment (*n* = 5). Data is presented as mean ± SEM and analysed by Two-way ANOVA and Bonferroni multiple comparison post hoc testing (**A**), One-way ANOVA and Bonferroni multiple comparison post hoc testing for parametric data (**E**) and Kruskal-Wallis test for non-parametric data (**B**, **C** and **D**). *p < 0.05, **p < 0.01, ***p < 0.001, ****p < 0.0001

On the other hand, we analyzed the body weight gain every other-week of mice fed with control diet or 45% HFD + 30% fructose in the drinking water (HFD + 30%Fr) during 10 weeks and then treated with either, vehicle or EDL6D for 10 more weeks (Fig. 6A). The results showed that HFD + 30%Fr fed mice had a significant increase in body weight gain when compared with control mice from week 6 until week 10 (Fig. 6A). Moreover, HFD + 30%Fr fed mice treated vehicle had a significant increase in body weight gain when compared with control mice from week 10 until the end of follow-up (20 weeks) (Fig. 6A). Interestingly, HFD + 30%Fr fed mice treated with EDL6D had also a significant increase in body weight gain when compared with control mice however these mice stop gaining weight at week 16 until the end of the study. This reduction in body weight gain when compared with HFD + 30%Fr fed mice treated vehicle did not reach statistical difference (Fig. 6A). Nevertheless, this is of importance since at the end of the 20-week period when total body differences were analyzed, the results showed that HFD + 30%Fr fed mice treated with vehicle had a significant increase in total body weight when compared with control mice, while EDL6D treated mice did not present increased body weight when compared with the control group (Fig. 6B). Importantly, EDL6D treatment did not alter food or water consumption when compared with vehicle treated mice (Supplementary Fig. 1B).

In addition, vehicle treated mice showed a significant increase in total liver weight when compared with control mice while EDL6D treatment was able to reduce significantly total liver weight to a similar weight of control mice, as with brown and epididymal adipose tissue (Table 2). Additionally, HFD + 30%Fr vehicle treated mice had an increased in non-specific liver damage measured as AST and ALT activity when compared to control mice, however the differences were only statistically significant for ALT (Fig. 6C-D). The EDL6D treatment was able to reduce the non-specific liver damage measured as AST and ALT activity when compared with vehicle treated mice however the differences were only statistically significant for AST (Fig. 6D). Importantly, EDL6D treatment did not affect testosterone serum levels when compared with vehicle treated mice (Fig. 6E).

**Table 2:** Tissue weight therapeutic study

Tissue	Control	Vehicle	EDL6D
Liver (g)	1.54 ± 0.07**	2.12 ± 0.29	1.67 ± 0.10*
Hepatic Index (%)	4.06 ± 0.11	4.71 ± 0.45	4.43 ± 0.11
Kideny (g)	0.45 ± 0.02	0.45 ± 0.01	0.46 ± 0.02
Spleen (g)	0.10 ± 0.01	0.12 ± 0.01	0.10 ± 0.003
Testis (g)	0.23 ± 0.004	0.23 ± 0.004	0.22 ± 0.004
Brown Adipose	0.25 ± 0.02***	0.50 ± 0.06	0.35 ± 0.04*
Epididymal Adipose	1.52 ± 0.16	1.6 ± 0.13	1.59 ± 0.11
Inguinal Adipose	0.57 ± 0.10**	1.46 ± 0.19	1.12 ± 0.17*

Liver, kidney, spleen, testis, brown adipose tissue and epididymal adipose tissue weight in control, vehicle and EDL6D treated mice at the end time point. Hepatic index percentage is calculated respect body weight at the end of the experiment (*n* = 8–10). Data is presented as mean ± SEM and analysed vs. Vehicle by One-way ANOVA and Bonferroni multiple comparison post hoc testing. *p < 0.05, **p < 0.01, ***p < 0.001, ****p < 0.0001

**Fig. 7:**
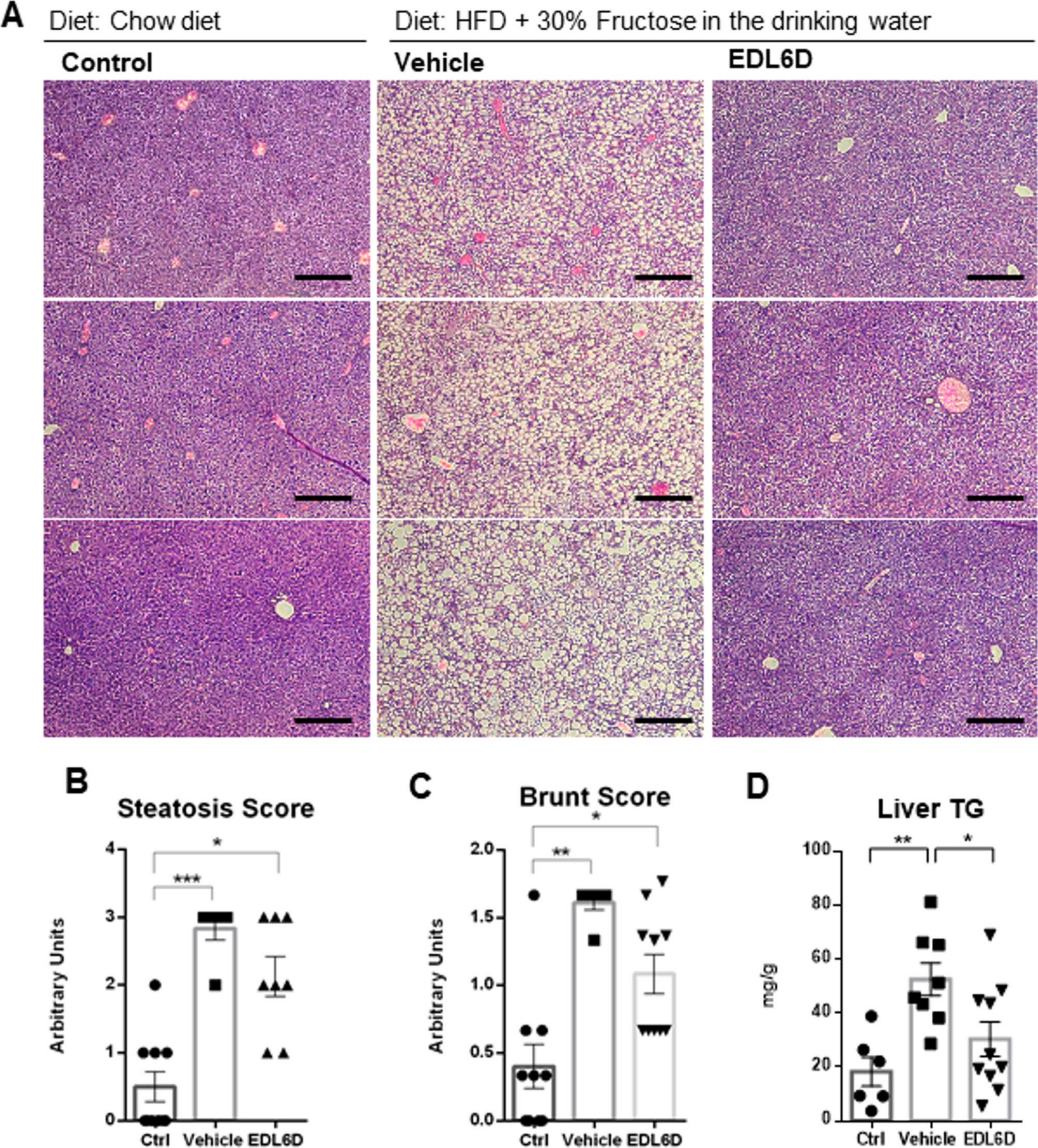
**A**. Representative images (10X – 100 μm scale bar) of hematoxylin/eosin staining of paraffin embedded liver tissue from Ctrl, Vehicle and EDL6D treated mice for 10 weeks under the different diet conditions, previously described (*n* = 8–10). **B**. Liver steatosis qualitative quantification (Steatosis Score) (from 0 until 4) and **C**. Liver Brunt Score (from 0 until 4) from Vehicle and EDL6D treated livers at the end time point performed by an external hepatologist (*n* = 8–10). **D**. Triacylglyceride quantification of liver lysates at the same time point (*n* = 8–10). Data is presented as mean ± SEM and analysed by One-way ANOVA and Bonferroni multiple comparison post hoc testing for parametric data (**D**) and Kruskal-Wallis test for non-parametric data (**B** and **C**). *p < 0.05, **p < 0.01, ***p < 0.001, ****p < 0.0001

The liver histological examination revealed that control mice had preserved hepatic architecture while HFD + 30%Fr vehicle treated mice developed a clear hepatic steatosis (Fig. 7A). Importantly, EDL6D treated mice showed a remarkable reduction in hepatic steatosis (Fig. 7A). Consistently, the EDL6D reduction in steatosis was again confirmed by a pathologist as previously described by means of steatosis and brunt scores (Fig. 7B-C). These results were further corroborated by measuring the TG content in the livers of control mice and HFD + 30%Fr mice treated with vehicle or EDL6D. The results showed that livers from mice fed with HFD + 30%Fr mice and treated with vehicle had significantly increased TG content when compared with control mice (Fig. 7D), while livers from mice fed with of HFD + 30%Fr plus EDL6D showed a significantly lower TG content when compared with mice fed with HFD + 30%Fr vehicle group (Fig. 7D). In addition, liver cholesterol levels did not show significant differences between groups (Supplementary Fig. 1D).

**Fig. 8:**
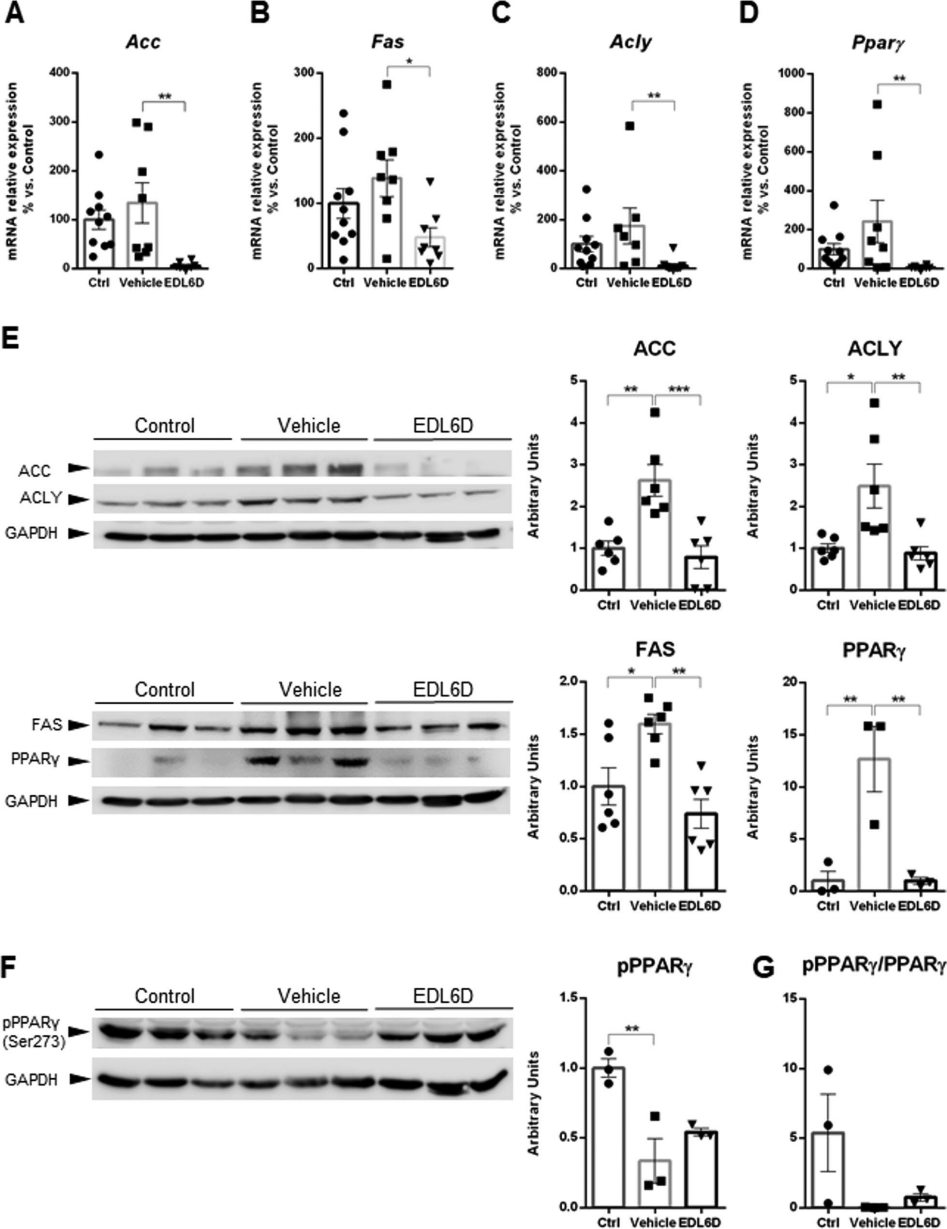
**A**. mRNA expression of *Acc*, *Acly*, *Fas* and Pparγ relative to control mice livers (*n* = 8–10) and **B**. representative western blot (WB) images of ACC, ACLY, FAS, PPARγ and pPPARγ and its respective quantifications compared to vehicle group (*n* = 3–6). **G**. pPPARγ/PPARγ ratio quantification (*n* = 3). Data is presented as mean ± SEM and analysed by One-way ANOVA and Bonferroni multiple comparison post hoc testing for parametric data (**B**) and Kruskal-Wallis test for non-parametric data (**A**, **C**, **D**, **E**, **F** and **G**). *p < 0.05, **p < 0.01, ***p < 0.001, ****p < 0.0001

**Fig. 9:**
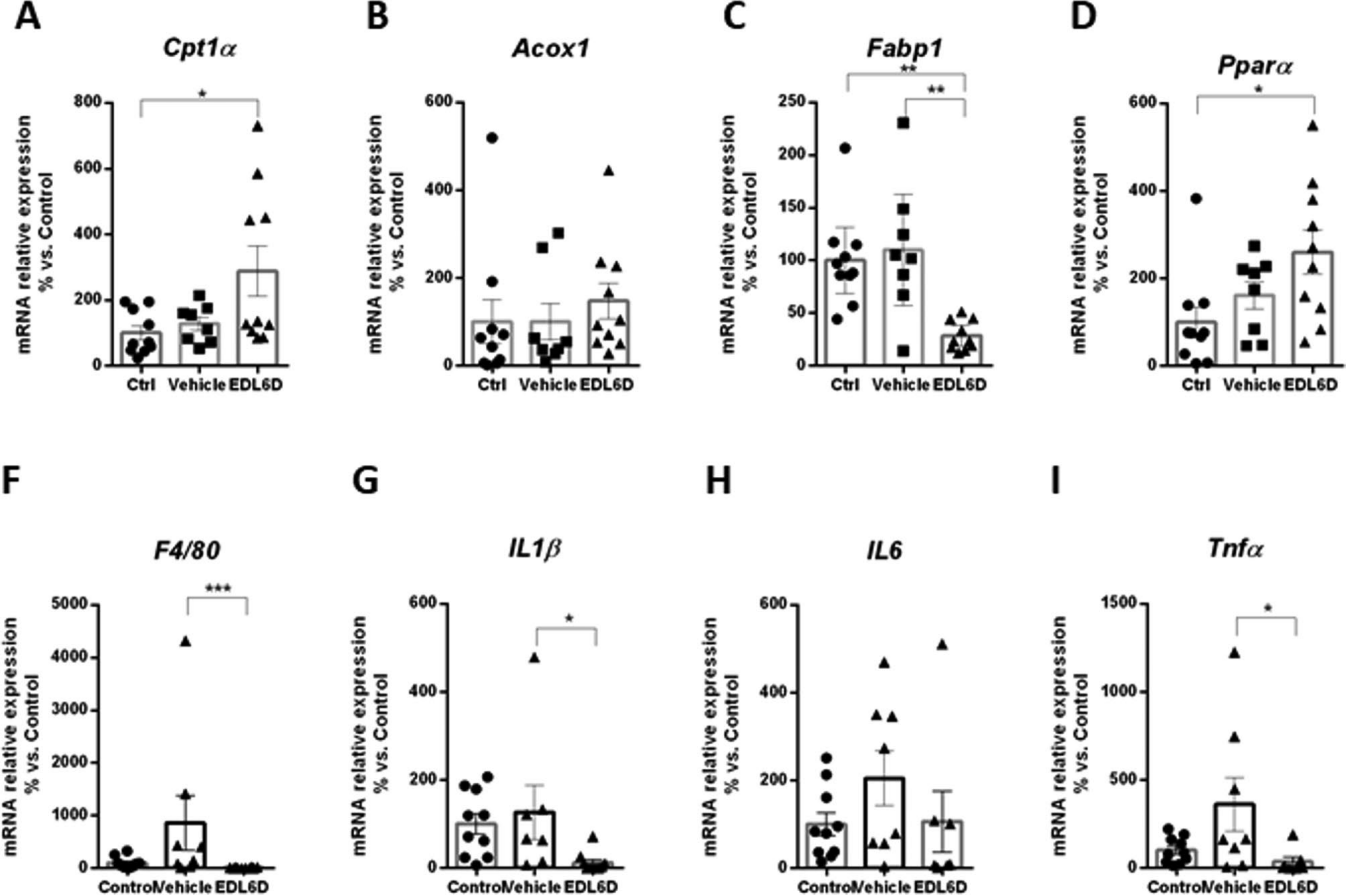
**A**-**K** mRNA expression of *Cpt1α Acox*,* Pparα*,* Fabp1*,* F4/80*, *Il1β*, *Il6* and *Tnfα*, relative to control treated mice livers (*n* = 8–10). Data is presented as mean ± SEM and analysed by One-way ANOVA and Bonferroni multiple comparison post hoc testing for parametric data (**C**) and Kruskal-Wallis test for non-parametric data (**A**, **B**, **D**, **F**,**G**, **H** and **I**). *p < 0.05, **p < 0.01, ***p < 0.001, ****p < 0.0001

Then we analyzed the hepatic levels of key genes involved in hepatic *de novo* lipogenesis (*Acc*, *Acly*, *Fas* and *Pparγ*), β-oxidation (*Cpt1α*, *Acox1*, *Pparα*), fatty acid uptake (*Fabp1*). Treatment with EDL6D was able to reduce the mRNA and protein levels of ACC, ACLY, FAS and PPARγ when compared with mice fed HFD + 30%Fr and treated with vehicle (Fig. 8A-E). EDL6D treatment tended to increase the PPARγ phosphorylated protein levels when compared with vehicle treated mice, however did not reach statistical significance (Fig. 8F), as shown as well by the pPPARγ/PPARγ ratio (Fig. 8G). The expression of the *Cpt1α* and *Acox1* revealed that EDL6D treatment upregulated the mRNA levels being significant for *Cpt1α* (Fig. 9A-B). Moreover, fatty acid uptake gene *Fabp1* was downregulated in the peptide treated mice compared to vehicle (Fig. 9).

Furthermore, analysis of the related inflammation gene expression revealed that mice treated with EDL6D were protected from the increase in mRNA levels of *F4/80*, and the proinflammatory cytokines *Il1β*, *Il6* and *Tnfα* induced by HFD + 30%Fr (Fig. 9F-I).

Finally, preliminary results from a dose range finding study showed that EDL6D treatment was able to reduce significantly the hepatic index in a dose dependent manner (2.5, 5, 10 and 20 mg/kg/day) (Supplementary Fig. 4). EDL6D treatment was able to reduce significantly the HFD + 30%Fr-induced increase in weight of liver, epididymal, inguinal and brown adipose depots (Supplementary Fig. 4). Moreover, histological analyses of H&E paraffin sections showed that control mice had preserved hepatic architecture while HFD + 30%Fr vehicle treated mice developed a clear hepatic steatosis (Supplementary Fig. 5). Importantly, EDL6D treated mice showed a remarkable reduction in hepatic steatosis at 5, 10 and 20 mg/kg/day in a dose-dependent manner (Supplementary Fig. 5).

### EDL6D treatment prevents fibrosis development

**Fig. 10:**
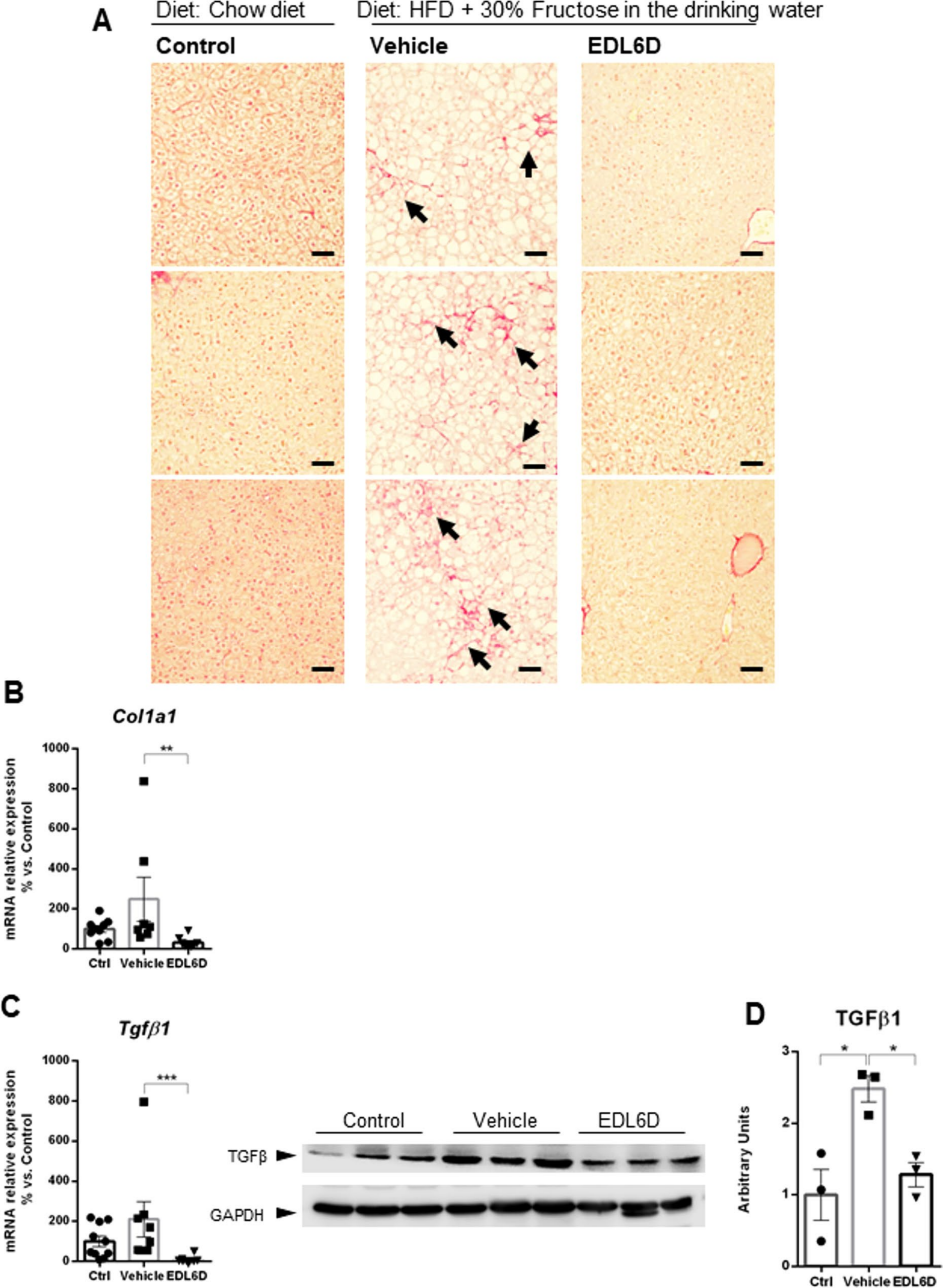
**A**. Representative images (10X – 100 μm scale bar) of Picro Sirius Red staining of paraffin embedded liver tissue from Ctrl, Vehicle and EDL6D treated mice for 10 weeks under the different diet conditions, previously described (*n* = 8–10). **B**. mRNA expression (*n* = 8–10) of Col1a1 and **B**. Transforming growth factor beta 1 (TGFβ1) mRNA and WB representative images (*n* = 3). Data is presented as mean ± SEM and analysed by One-way ANOVA and Bonferroni multiple comparison post hoc testing for parametric data (**B** and **C**) and Kruskal-Wallis test for non-parametric data (**D**). *p < 0.05, **p < 0.01, ***p < 0.001, ****p < 0.0001

Liver histological examination of fibrosis by picro sirius red staining was performed at the end of follow-up (20 weeks). The results revealed that control mice had no abnormal collagen deposition while 3 out of 10 mice fed with HFD + 30%Fr and treated with vehicle showed mild fibrosis development as evidenced by having abnormal collagen deposition (Fig. 10A). Notably, none of the EDL6D treated mice showed abnormal collagen deposition (Fig. 10A). The protective effect of EDL6D in fibrosis development was confirmed by measuring *Col1a1* expression. In this regard we found that EDL6D treatment was able to reduce significantly the *Col1a1* mRNA levels when compared with vehicle treated mice (Fig. 10B). In addition, HFD + 30%Fr vehicle treated mice showed a significant increase in TFGβ-1 mRNA and protein levels when compared with control mice, which was completely abrogated by EDL6D treatment (Fig. 10C-D).

## Discussion

There are several associated risk factors for MASLD that include T2D, obesity and increased hepatic lipogenesis among others [29]. It is worth mentioning, that subjects suffering from the above diseases are all characterized by having low plasma levels of SHBG, and in fact circulating SHBG levels are also low in MASLD and MASH [7, 30–34]. In addition, epidemiological studies have shown that low circulating SHBG is a risk factor for developing T2D and cardiovascular disease [35–39]. During the last decade we have demonstrated that rather than a merely biomarker, low hepatic production of SHBG plasma levels could play a role in the development and/or progression of MASLD and obesity [24]. In this regard, we have shown that SHBG reduces lipid fat accumulation by inhibiting lipogenesis and inducing lipolysis [24, 40], which pointed-out SHBG as a new therapeutic target to develop novel treatments against these metabolic disorders. In the current proof-of-concept study, we provide evidence that EDL6D, a bioactive peptide that phenocopies SHBG-associated metabolic effects inhibit lipogenesis. To do so, we used an in vitro approach using the HepG2 cells treated daily with 30 mM fructose, which has been shown to increase palmitate production [9]. In addition, we have also demonstrated that EDL6D prevents and regress hepatic lipid accumulation with an associated anti-inflammatory and antifibrotic activity in a well-established mouse model of MASLD.

In previous reports, we have shown that SHBG overexpression reduces hepatic steatosis in two different mouse models of MASLD, a genetically induced (*SHBG-C57BL/ksJ-db/db* transgenic mice) mouse model and a high carbohydrate diet induced (human *SHBG* transgenic mice) mouse model. Moreover, we provided evidence that SHBG has a direct effect on hepatocytes independently of sex steroids [24]. Here, we report that EDL6D treatment was able to prevent the development of MASLD induced by high fat diet and 30% fructose in the drinking water (HFD + 30%Fr) during 8 weeks by reducing hepatomegaly and hepatic steatosis via inhibiting hepatic lipogenesis. Furthermore, HFD feeding induced a significant increase in hepatic TG when compared with control mice as described previously [41], remarkably EDL6D treatment showed a profound therapeutic effect by regressing TG accumulation when compared with HFD vehicle treated mice.

Several other compounds have shown beneficial effects in reducing hepatic steatosis in mouse models of MASLD. Among them GLP-1R agonists have shown encouraging results [42–44]; however, the underlying mechanisms seem more related to reduction of body weight and insulin resistance than a direct liver action and they have failed in preventing or arresting fibrosis [45, 46]. In contrast, our results showed that EDL6D reduction of hepatic steatosis was not mediated by reducing food intake, since EDL6D treatment did not change mouse food intake in the preventive or therapeutic studies. However, a weight loss also occurs, thus targeting extrahepatic metabolic pathways that participate in MASDH development. In addition, and more importantly, the EDL6D preventive and therapeutic effects were mechanistically mediated by a significant reduction in mRNA and protein levels of key liver lipogenic enzymes (ACLY, ACC and FAS) as well as PPARγ, a crucial lipogenic transcription factor compared to vehicle treated mice, as it has been previously reported for SHBG [24]. Therefore, EDL6D exerts a dual action via systemic effect inducing weight loss and an essential direct effect inhibiting hepatic lipogenesis. Preliminary data of a dose range finding study showed that EDL6D treatment was able to reduce significantly the hepatic index, liver weight and hepatic steatosis in a dose dependent manner, and among the doses tested 5 mg/kg/day was the minimum efficacy dose. Another important action of EDL6D is the intrahepatic anti-inflammatory action. In this regard, we have found a significant downregulation of mRNA levels of *F4/80*, a marker of macrophage infiltration, and proinflammatory cytokines such as interleukin 1 beta (*Il1β*) and tumor necrosis factor alpha (*Tnfα*).

Remarkably, in our 20-week therapeutic study, 30% of the mice fed with HFD + 30%Fr treated with vehicle showed development of very mild fibrosis after histological examination, but none of the EDL6D treated mice showed evidence of it. It is well known that TGF-β1 is a key inflammatory cytokine involved in the progression from MASLD to MASH and fibrosis [16–18] and previous studies have demonstrated that hepatic TGF-β1 expression is increased in MASH patients with fibrosis [19–22]. TGF-β1-mediated signalling is a key stimulator of collagen production [47] and excessive collagen production causes organ fibrosis [48, 49]. This anti-fibrotic effect of EDL6D was further supported by its capacity to downregulate *Col1a1* and *Tgf-β1* mRNA levels when compared with vehicle treated mice. These encouraging results position EDL6D as new preclinical lead compound not only for the prevention and/or treatment of MASLD, but it could also prevent liver fibrosis development.

Until recently, the only recommended treatment for MASLD was lifestyle modification, which included diet and exercise. In March 2024, Resmetirom was FDA-approved becoming the first medication approved for treating MASLD and MASH. Resmetirom is an orally administered, liver-targeted thyroid hormone receptor-β (THRβ) selective agonist which activates THRβ, a nuclear hormone receptor predominantly expressed in the liver that acts as a regulator several metabolic pathways in this organ. However, the exact mechanisms through which activation of THRβ improves MASH are still unclear. In a phase III randomized controlled trial, 966 patients with mild-to-moderate MASH were randomly assigned to once-daily oral treatment with 80 mg or 100 mg resmetirom or placebo for 52 weeks. MASH resolution, defined by histological analysis of biopsy samples, with no worsening of fibrosis, was observed in 25.9% of the 80 mg group and 29.9% of the 100 mg group, compared to 9.7% in the placebo group (*P* < 0.001). Treatment was associated with fibrosis improvement by at least one stage with no worsening MASH activity score in 25% of resmetirom recipients versus just under 15% in the placebo group [50]. It is worth mentioning that at least in terms of reduction of hepatic fat, the beneficial effects of resmetirom have been directly related to the increase of plasma levels of SHBG [51, 52]. This is a crucial issue because SHBG is considered as a “measurement of exposure” to resmetirom and only those patients with ≥ 120% increase from baseline in SHBG could be considered as responders [51]. Unfortunately, data regarding SHBG were not presented the clinical trial aimed at assessed the effectiveness of resmetirom in MASH with liver fibrosis [50]. Our findings suggest that rather than being a biomarker of response to resmetirom, SHBG is an essential mediator of its beneficial effects in both MASLD and MASH. In fact, we reported several years ago that thyroid hormones increase SHBG production indirectly by increasing HNF-4alpha gene expression, and by reducing cellular palmitate levels that further contribute to increased HNF-4alpha levels in hepatocytes [50]. By targeting the three key conditions involved in MASH: fat accumulation, inflammation and fibrosis development, EDL6D can be contemplated as an excellent therapeutic strategy. In addition, the direct hepatic EDL6D actions could overcome the relatively low effectiveness rate observed with resmetirom, which might be attributed to the heterogenicity in the SHBG enhancement mediated by THRβ. However, specific experimental research studies to confirm this hypothesis, as well as clinical trials to test EDL6D safety and effectiveness are needed.

A growing number of emerging therapies for MASLD and MASH are currently targeting key metabolic regulators, such as FGF21 analogues and enzymes involved in lipid metabolism adding mechanistic diversity to the therapeutic pipeline. In addition, increasing attention is being directed toward nanotechnology-based delivery strategies to overcome several limitations of therapeutic peptides, such as poor stability and limited tissue specificity. Recent preclinical studies have demonstrated that lipid nanocapsules and polymeric nanocarriers improved peptide bioavailability with better liver targeting which resulted in an increase in therapeutic efficacy in MASLD models. In this context, the use of biocompatible and bioactive materials has gained interest for their favourable safety and functional profiles [26, 27]. Future perspective includes the combination of peptide-based therapies with emerging green or biocompatible nanomaterials (e.g. biodegradable liposomes and polymeric nanoparticles) which represents a promising strategy to improve specificity, minimize undesired secondary effects by controlling drug release, which will support a more efficient translation toward clinical application in MASLD.

Despite the strengths of this proof-of-concept study, several limitations should be acknowledged. First, all in vivo experiments were performed exclusively in male mice and, therefore, potential sex-specific responses to the EDL6D peptide remain to be determined. Second, the preventive study also did not include a chow-fed control group, which restricts direct comparison with physiological baseline conditions. Third, the lack of pair-feeding, indirect calorimetry, and physical activity monitoring prevents clear differentiation between direct hepatic effects of EDL6D and secondary improvements resulting from changes in systemic energy balance or metabolism. Finally, although EDL6D effectively prevented the development of mild fibrosis, the antifibrotic effects were evaluated only at an early stage. Therefore, further investigation incorporating additional fibrosis markers, including α-SMA, are required to determine whether EDL6D treatment can halt or reverse established fibrosis and to elucidate the mechanisms underlying its antifibrotic actions.

In summary, we have shown that EDL6D, a bioactive peptide phenocopying SHBG reduces de novo lipogenesis by reducing hepatic levels of ACC, ACLY, FAS and PPARγ, exerts an important anti-inflammatory effect by reducing expression of F4/80, a marker of macrophage infiltration, and proinflammatory cytokines such as IL1β, and TNFα. Moreover, EDL6D appears to modestly inhibit fibrosis development by reducing hepatic expression of Col1a1 and Tgf-β1 in MASLD mouse models. These results position EDL6D as promising new preclinical lead compound to develop new treatments to prevent and treat MASLD development and arrest its progression.

## Methods

### Peptide EDL6D

20-amino acids peptide EDL6D (EP2024/065136) has been obtained from ChemPeptide Services (Shanghai, China) and synthesis batch coded P220610-GB967738 has been released under the Certificate of Analysis issued 11 July 2022.

### Cell culture experiments

Cell culture reagents were purchased from Life Technologies Inc (Invitrogen SA, Barcelona, Spain). HepG2 hepatoblastoma cells (catalog no. HB-8065; ATCC, LGC Standards SLU, Barcelona, Spain) were maintained in Dulbecco’s modified Eagle’s medium (DMEM) supplemented with 10% fetal bovine serum (FBS) and antibiotics (100 U/ml penicillin and 100 µg/ml streptomycin), at 37 °C in a humidified atmosphere containing 5% CO_2_.

For experiments, HepG2 cells were cultured to 70–80% confluence in DMEM medium supplemented with 10% FBS and antibiotics. HepG2 cells were treated daily with 30 mM fructose in the absence or presence of EDL6D (40 µg/ml) for 24 h. After treatments, cells were scraped for RNA and protein analysis. All experiments were performed using six-well plates in triplicate and in at least three different experiments.

### In vivo experiments

C57BL/6 N wild type male mice were purchased from Charles River, Spain. Mice were maintained in ventilated cages with group housing (2–4 per cage), on a 12 h light/12 h dark cycle, in a temperature-controlled (20–24 °C) facility, with *ad libitum* access to food and water. Experimental procedures were approved by the Institutional Animal Use Subcommittees of Vall Hebron University Hospital Research Institute and the Universitat Autònoma de Barcelona (registry no. 45/13, Animal Experimental Ethics Committee).

#### Preventive study

Five-week-old C57BL/6J wild type mice were fed either a chow (R105-25, Safe Diets) or a 45% high fat diet (TD.190179, Envigo) supplemented with 30% fructose in the drinking water for a period of 8 weeks to induce MASLD development. In parallel, vehicle (*n* = 7) and EDL6D (20 mg/kg) (*n* = 9) were daily administered subcutaneously (Supplementary Fig. 2). Mice weight was monitored every week. At the end of the study, random fed mice were euthanised by cervical dislocation blood samples were taken by saphenous vein and tissues were harvested, weighed and stored at -80 °C until further processing. Liver samples were grinded using a mortar and pestle in liquid nitrogen. Liver was further homogenized for RNA isolation, protein analysis or triglycerides quantification.

#### Therapeutic study

Five-week-old C57BL/6J wild type mice were fed either a chow (R105-25, Safe Diets) (*n* = 8) or a 45% high fat diet (TD.190179, Envigo) supplemented with 30% fructose in the drinking water (*n* = 18) for a period of 10 weeks. After 10 weeks, diet induced-MASLD mice were treated with a daily subcutaneous injection of vehicle (*n* = 8) or EDL6D (20 mg/kg) (*n* = 10) (Supplementary Fig. 2). As in the previous study, mice weight was monitored every two weeks. Blood and liver samples were harvested and processed as described for the preventive study.

#### Therapeutic dose range finding study

Five-week-old C57BL/6J wild type mice were fed either a chow (R105-25, Safe Diets) (*n* = 10) or a 45% high fat diet (TD.190179, Envigo) supplemented with 30% fructose in the drinking water (*n* = 50) for a period of 10 weeks. After 10 weeks, diet induced-MASLD mice were treated with a daily subcutaneous injection of vehicle (*n* = 10) or EDL6D (2.5, 5, 10 and 20 mg/kg) (*n* = 10 each). As in the previous studies, mice weight was monitored every two weeks. Blood and liver samples were harvested and processed as described for the previous studies.

### Histology

For morphological studies, livers were fixed in 4% paraformaldehyde for 24 h and embedded in paraffin. Serial 5-µm-thick sections were processed for hematoxylin/eosin (PRC/R/42, PRC/66/1, Pioneer Research Chemical) staining and Picrosirius red staining as previously described (16). These sections were examined with and imaged using an inverted microscopy (Eclipse Ts3R-FL, Nikon).

### RNA analysis

Total RNA was extracted from pulverized liver or HepG2 scraped cells form 6-well plate wells using 1 ml NZYol (Nzytech). Reverse transcription (RT) was performed at 42 °C, for 50 min using 3 µg of total RNA and 200 U of Superscript II together with oligo-dT primers and reagents provided by Invitrogen. Quantitative RT-PCR was carried out with SYBR Green PCR master mix (Invitrogen SA) on the QuantStudio 7 Pro PCR system (Applied Biosystems) in a 12 µl volume using 2 µl cDNA, 1:10 diluted. All reactions were carried out in duplicate and Ct values were obtained. Relative differences in the gene expression were normalized to expression levels of housekeeping gene 18 S or HPRT using the deltaCt analysis method. Primer sequences are shown in table Supplementary Table 1.

### Triglyceride and cholesterol quantification

Liver was homogenized in 1 ml 5% NP-40 in water and triglycerides were extracted and quantified using a colorimetric Triglyceride Assay Kit (Ab65336, Abcam) following the manufacturer’s instructions. Cholesterol was measured using cholesterol Liquid, Trinder Method-Endpoint (CL21-400 S, FAR diagnostics) following manufacturer’s instructions.

### Western blot analysis

HepG2 cell lysates were obtained from 6-well plate wells after incubating at 4 °C o/n with 1 ml RIPA buffer with Complete protease inhibitor cocktail (Roche Diagnostics). On the other hand, approximately 80–100 mg of pulverized liver tissue was homogenized in RIPA buffer with Complete protease inhibitor cocktail (Roche Diagnostics). Protein extracts were used for western blotting with antibodies against ACC (ab45174, Abcam), FAS (ab128856, Abcam), ACLY (3661, Cell Signaling), PPARγ (2443, Cell Signaling), Col1A1 (91144, Cell Signaling), TGFβ-1 (ab215175, Abcam), pPPARγ (Ser273) (bs-4888R, Bioss) and GAPDH (2118, Cell Signaling). Specific antibody-antigen complexes were identified using an HRP-labeled goat anti-rabbit IgG (P0448, Dako, Glostrup, Denmark), rabbit anti-mouse IgG (P0260, Dako) or rabbit anti-goat IgG (P0449, Dako) and Immobilon chemiluminescent HRP substrate (Merck-Millipore, Darmstadt, Germany) was used for detection by exposure using Odissey Fc instrument. Band intensity analysis was performed by ImageJ 1.53k.

### Serum analysis

Serum samples from the in vivo studies were used to measure aspartate aminotransferase (AST) and alanine aminotransferase (ALT) activity using the AST Activity Assay Kit (MAK055, Sigma-Aldrich) and the ALT Assay kit (MAK052, Sigma-Aldrich) following the manufacturer’s instructions. Serum testosterone levels were assessed using the Testosterone ELISA kit (DE1559, Demeditec Diagnostics GmbH).

### Statistical analysis

Quantitative data is reported as mean ± SEM. Prior to statistical analysis, data distributions were assessed for normality using the Shapiro–Wilk test. Data that met these assumptions were analysed using Student’s t test or Mann-Whitney (for two-group comparisons), one-way ANOVA (for single-factor comparisons involving more than two groups) for parametric and Kruskal-Wallis test for non-parametric data, or two-way ANOVA (for analyses involving two independent variables), as appropriate and indicated in the Figure legends, using GraphPad Prism software (GraphPad 6.01 for Windows, California). Statistical significance was defined as *p* < 0.05 (*p < 0.05, **p < 0.01, ***p < 0.001, ****p < 0.0001).

## Electronic Supplementary Material

Below is the link to the electronic supplementary material.

Supplementary Material 1

Supplementary Material 2

Supplementary Material 3

Supplementary Material 4

Supplementary Material 5

## Data Availability

The datasets generated and/or analyzed during the current study are available from the corresponding author on reasonable request.
