# Supplementary figures and images for: EDL6D, a bioactive peptide phenocopying SHBG-associated metabolic effects: a new preclinical lead compound for treating the metabolic dysfunction-associated fatty liver disease
Source: J Transl Med. 2026 May 16;24:883. doi: 10.1186/s12967-026-08305-9 (PMC13360854; doi:10.1186/s12967-026-08305-9)

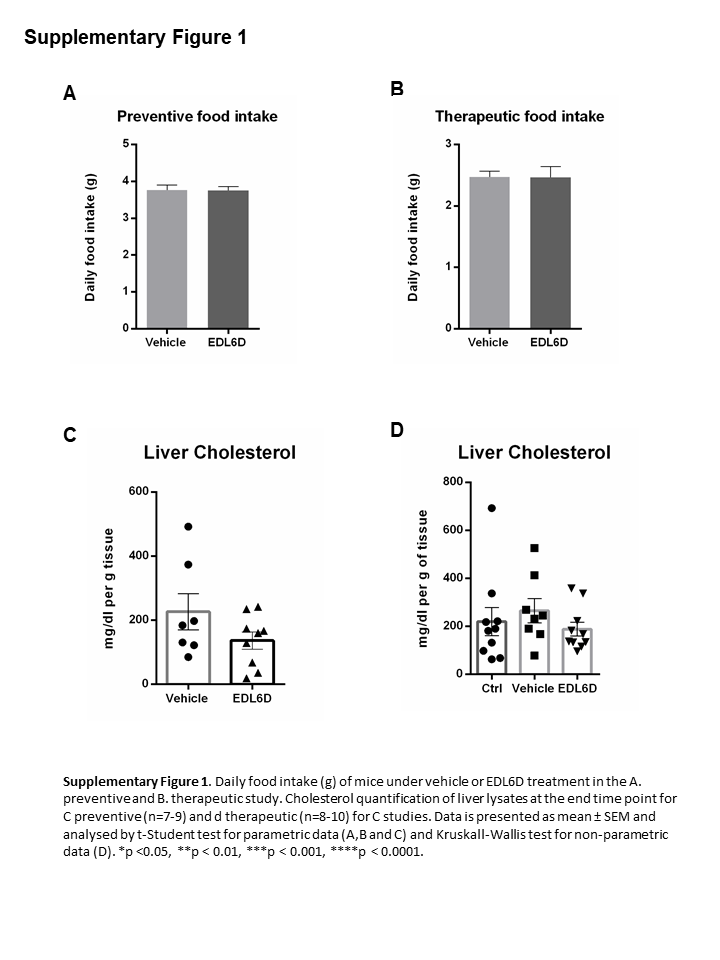

Supplement: Supplementary file 1 — Supplementary Material 1 [file 12967_2026_8305_MOESM1_ESM.tif]

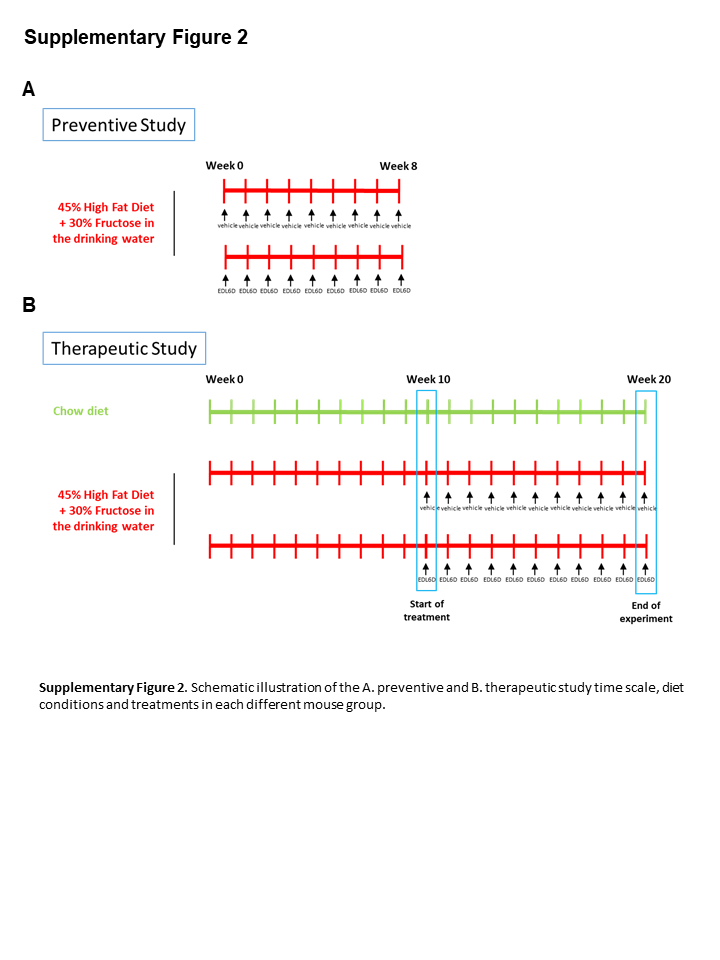

Supplement: Supplementary file 2 — Supplementary Material 2 [file 12967_2026_8305_MOESM2_ESM.tif]

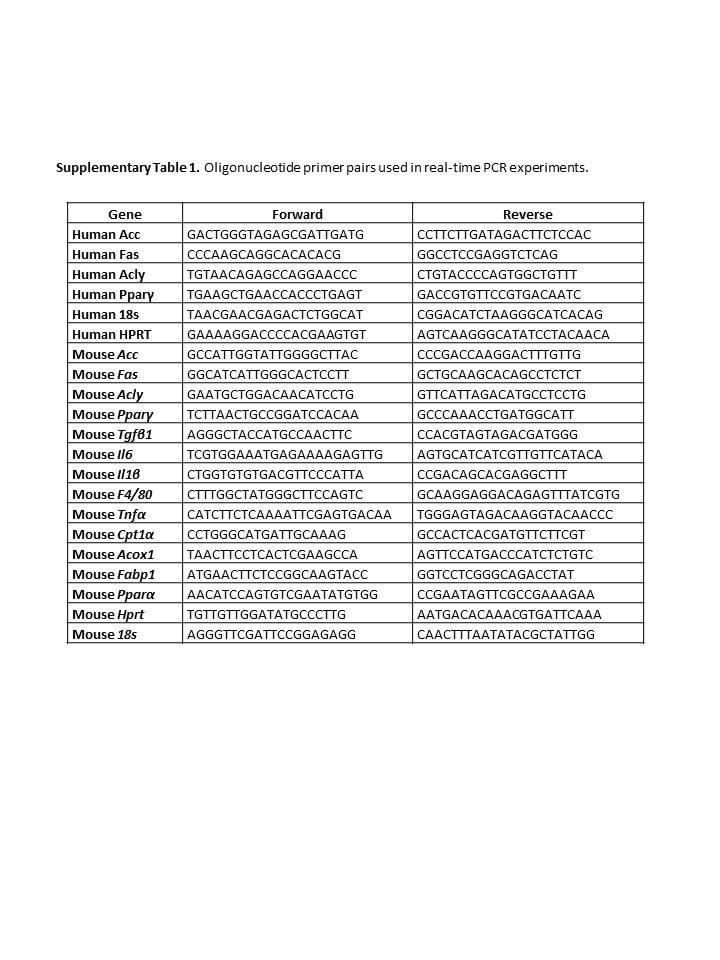

Supplement: Supplementary file 3 — Supplementary Material 3 [file 12967_2026_8305_MOESM3_ESM.tif]

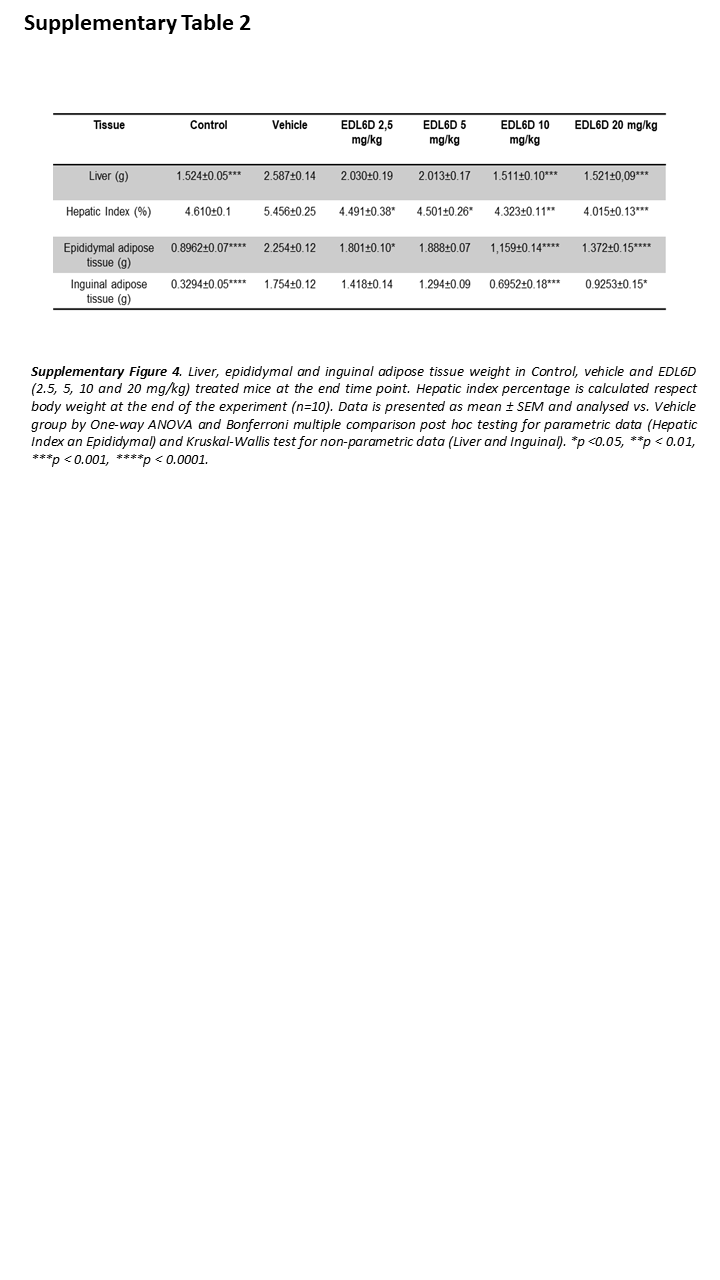

Supplement: Supplementary file 4 — Supplementary Material 4 [file 12967_2026_8305_MOESM4_ESM.tif]

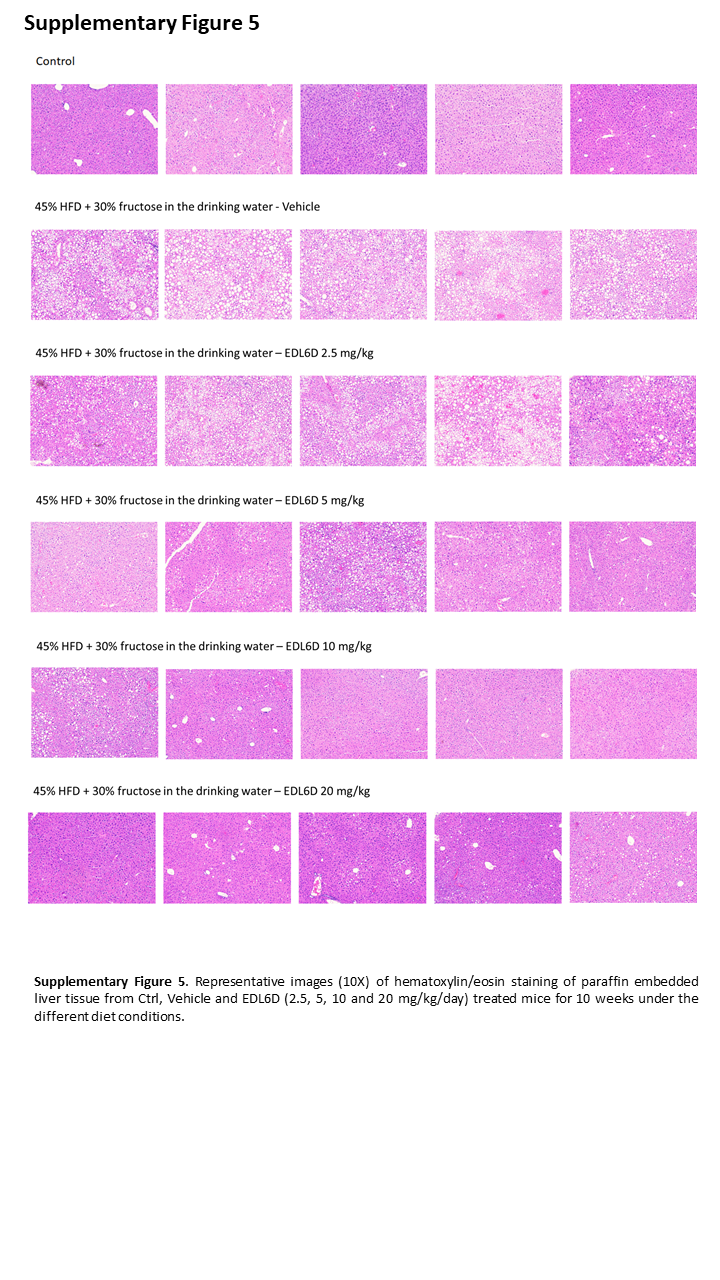

Supplement: Supplementary file 5 — Supplementary Material 5 [file 12967_2026_8305_MOESM5_ESM.tif]
